# A cost analysis of robotic vs. video-assisted thoracic surgery: The impact of the learning curve and the COVID-19 pandemic

**DOI:** 10.3389/fsurg.2023.1123329

**Published:** 2023-04-25

**Authors:** Oliver J. Harrison, Alessandro Maraschi, Tom Routledge, Savvas Lampridis, Corinne LeReun, Andrea Bille

**Affiliations:** ^1^ Thoracic Surgery Department, Guys Hospital, London, United Kingdom; ^2^ Department of Thoracic Surgery, University Hospital Southampton, Southampton, United Kingdom; ^3^ Conseil en Analyse Statistique et Modélisation économique, Sainte-Anne, Guadeloupe

**Keywords:** lung cancer, robot—assisted thoracic surgery, video assisted thoracic surgery, cost analyses, COVID—19, learning curve

## Abstract

**Introduction:**

Robot-assisted thoracoscopic surgery (RATS) is an alternative to video-assessed thoracoscopic surgery (VATS) for the treatment of lung cancer but concern exists regarding the high associated costs. The COVID-19 pandemic added further financial pressure to healthcare systems. This study investigated the impact of the learning curve on the cost-effectiveness of RATS lung resection and the financial impact of the COVID-19 pandemic on a RATS program.

**Methods:**

Patients undergoing RATS lung resection between January 2017 and December 2020 were prospectively followed. A matched cohort of VATS cases were analyzed in parallel. The first 100 and most recent 100 RATS cases performed at our institution were compared to assess the learning curve. Cases performed before and after March 2020 were compared to assess the impact of the COVID-19 pandemic. A comprehensive cost analysis of multiple theatre and postoperative data points was performed using Stata statistics package (v14.2).

**Results:**

365 RATS cases were included. Median cost per procedure was £7,167 and theatre cost accounted for 70%. Major contributing factors to overall cost were operative time and postoperative length of stay. Cost per case was £640 less after passing the learning curve (*p* < 0.001) largely due to reduced operative time. Comparison of a post-learning curve RATS subgroup matched to 101 VATS cases revealed no significant difference in theatre costs between the two techniques. Overall cost of RATS lung resections performed before and during the COVID-19 pandemic were not significantly different. However, theatre costs were significantly cheaper (£620/case; *p* < 0.001) and postoperative costs were significantly more expensive (£1,221/case; *p* = 0.018) during the pandemic.

**Discussion:**

Passing the learning curve is associated with a significant reduction in the theatre costs associated with RATS lung resection and is comparable with the cost of VATS. This study may underestimate the true cost benefit of passing the learning curve due to the effect of the COVID-19 pandemic on theatre costs. The COVID-19 pandemic made RATS lung resection more expensive due to prolonged hospital stay and increased readmission rate. The present study offers some evidence that the initial increased costs associated with RATS lung resection may be gradually offset as a program progresses.

## 1. Introduction

Surgery remains the gold standard treatment for early stage lung cancer a with 5-year survival rate of 90%. In the 1990s, video-assisted thoracoscopic surgery (VATS) was introduced and became the standard surgical approach offered for the treatment of early stage lung cancer (1). More than 20 years after its introduction, the VIOLET trial and other randomized studies have demonstrated that VATS is associated with shorter length of hospital stay and fewer complications compared to open surgery (2, 3).

Concurrently, over the last two decades robot-assisted thoracoscopic surgery (RATS) has evolved into thoracic surgical practice. Several retrospective studies have demonstrated the potential benefits of RATS over VATS and open surgery including improved the lymph node clearance, fewer conversions and reduced length of hospital stay (4–6). However, there are concerns regarding the increased costs associated with RATS, particularly vs. VATS, which have been further exacerbated by studies demonstrating similar outcomes between RATS and VATS (7–9)*.* Due to a lack of randomized trials, there is still an open debate regarding the best and most cost-effective minimally invasive approach in the treatment of lung neoplasms.

In current times more than ever there is intense scrutiny on postoperative outcomes and the cost-effectiveness of new technologies and devices. A cost analysis is hugely important to better understand if the potential benefits of RATS surgery can be cost-effective for patients and national health systems. Upfront costs of purchasing a robotic system are steep and the additional operative time taken to get over the learning curve absorbs further resources. Indeed, evidence from urological surgery suggests that learning robotic surgery is costly, but these costs can be attenuated by high volume exposure to flatten the learning curve (10). There is certainly a desire among the robotic surgical community to explore this concept further (11).

Our center is currently the highest volume thoracic unit for lung cancer resection in the UK National Health Service with over 70% of cases performed minimally invasively (12). RATS was introduced in 2017 and our center is now one of the highest volume robotic thoracic units in Europe. Our impression is that after completing the learning curve, RATS may have similar or even improved cost-effectiveness compared to VATS. Similarly, in March 2020 the COVID-19 pandemic began to have a huge impact on the provision of lung cancer care in the UK.

The primary aim of this study was to determine the impact of the learning curve on the cost-effectiveness of RATS vs. VATS lung resection. The secondary aim was to assess impact of the COVID-19 pandemic on postoperative costs in patients who had minimally invasive lung cancer surgery and assess if RATS could help to contain some of the increased cost burden inflicted by the COVID-19 pandemic.

## 2. Materials and methods

### 2.1. Patient selection

This study was conducted in accordance with the Declaration of Helsinki. Given the retrospective nature of the study the need for written consent was waivered by the institutional review board. Patients who underwent RATS lobar or sublobar lung resection for neoplasm between January 2017 and December 2020 were identified and included in the analysis. Patients who had undergone prior VATS or thoracotomy, a pneumonectomy or chest wall resection were excluded. VATS lobar and sublobar resections performed between April 2015 to December 2020 were used as comparison group. Patient demographics, clinical data and outcomes were accessed from the Thoracic Surgery department prospective database (IRB approval number: 13197, January 2021). Complications were classified according to the Clavien-Dindo scale (13). Operative time was measured from skin incision to the completion of wound closure. Requirement for blood transfusion and rate of conversion to open were also captured. All RATS procedures were performed by two high volume robotic surgeons using the same technique. VATS procedures were performed by one of the two surgeons with a high-volume experience in VATS lobectomy. Cost calculations were performed using our institutional cost codes.

To evaluate the impact of learning curve on cost, patients were grouped into the first 100 RATS cases performed and most recent 100 RATS cases performed by the two surgeons combined. To evaluate the impact of COVID-19 on cost, patients undergoing operations before March 2020 vs. those performed during and after March 2020 were analyzed separately. A comparative cost analysis of VATS and RATS was also performed using two subgroups matched on performance status and subtype of procedure, which were all performed by a single surgeon.

### 2.2. Surgical technique

All RATS cases were performed using the Da Vinci Model Xi (Intuitive Surgical, USA) using a 4-arm approach: 2 × 8 mm ports, 2 × 12 mm ports and 1 × 15 mm assistant port. Insufflation of CO_2_ was used at a pressure of 6 mmHg in all cases. Fenestrated bipolar forceps, permanent cautery spatula and Cadiere forceps were the standard instruments used together with the SureForm™ 45 EndoWrist stapler. VATS lung resections were performed using a 3-port approach according to the Mckenna Technique (14). Manual ENdoGIA staplers (Covidien/Medtronic, UK) were used in all cases. Intercostal nerve block was performed for each case and a single 28 Fr chest drain was positioned at the end and suction (-2 KPa) was applied for the first 24 h.

### 2.3. Cost analysis

The primary financial outcome was to analyse the direct cost related to RATS surgery and to compare this with VATS costs. Total direct costs were defined as the cost of specific items used in the patient care intraoperatively. All unit costs of consumables were provided by Intuitive and Guy's and St Thomas NHS Foundation trust. The cost of all reusable instruments, devices and staplers were included in the analysis.

Operating theatre time (cost per minute) was based on NHS Improvement data (15). The cost of blood transfusion and readmission were based on the NICE Costing Statement (16). The cost of hospital stay on the ward and in the intensive care unit (ICU) were based on the National Schedule of NHS costs 2018–2019 (17). The cost of complications was based on previously published figures (18, 19).

The postoperative management was identical for RATS and VATS patients and therefore not accounted for in the cost-analysis. Costs related to 30-day readmission were also included. Capital costs of the robotic systems VATS sets and vision stack were not included in this analysis. A full list of costing and sources can be found in Supplementary Table S1.

### 2.4. Statistical analysis

Categorical variables were presented as frequencies and percentage and were analyzed using Chi-squared tests. Continuous variables were reported as means ± standard deviations and median. Mean and median costs were calculated by multiplying the average resources used by each patient by the corresponding unit costs. Costs were compared between groups using Mann-Whitney *U* tests. When comparing the cost of RATS and VATS procedures, the first 100 cases (learning curve cases) were excluded. Data analysis was performed with Stata (v14.2; StataCorp LLC, USA).

## 3. Results

365 consecutive RATS lung resections were included in the analysis: 132 (36.2%) patients were male with a median age of 71. 355 (97.8%) patients underwent an anatomical lung resection. Patient characteristics are summarized in Table 1. The median operating time was 127 min. An R0 resection was achieved in 362 cases (99%). In 15 cases the robotic procedure was converted to open due to oncological reasons (*n* = 9) or bleeding (*n* = 6). Only 6 patients required a blood transfusion. The median time to chest drain removal was 2 days and median length of hospital stay was 5 days. 36.8% of patients experienced postoperative complications. Nineteen patients (5.2%) required an unplanned ICU admission. A detailed report of complications is given in Supplementary Table S2.

**Table 1:** Patient characteristics of the RATS cohort.

	RATS (*N* = 365)
Age (years)*	69.7 +/− 9.8 (71)
Gender (*n*)	
Male	132 (36.2%)
Female	233 (63.8%)
Performance Status (ECOG; *n*)	
0	121 (33.2%)
1	176 (48.2%)
2	68 (18.6%)
Procedure (*n*)	
Lobectomy	278 (76.2%)
Bilobectomy	7 (1.9%)
Segmentectomy	70 (19.2%)
Wedge resection	10 (2.7%)
Histology (*n*)	
Adenocarcinoma	226 (61.9%)
Squamous cell carcinoma	49 (13.4%)
Other NSCLC	4 (1.1%)
Metastasis	46 (12.6%)
Carcinoid	33 (9.0%)
Other	6 (1.6%)
Adenocarcinoma, SCLC	1 (0.3%)
Duration of chest drain (days)*	3.7 +/− 5.0 (2)
Length of stay (days)*	6.7 +/− 8.4 (5)
Planned ICU admission	
Number of patients	16 (4.4%)
Days in ICU*	1.8 +/− 1.5 (1)
Unplanned ICU admission	
Number of patients	19 (5.2%)
Days in ICU*	9.6 +/− 10.0 (6)
Complications (Clavien-Dindo grade; *n*)	
*0*	233 (63.8%)
*I*	55 (15.1%)
*II*	50 (13.7%)
*III*	17 (4.7%)
*IV*	6 (1.6%)
*V*	4 (1.1%)

* mean ± standard deviation (median). ECOG: Eastern Cooperative Oncology Group; ICU: Intensive care unit; NSCLC: Non-small cell lung cancer; SCLC: Small cell lung cancer.

### 3.1. Cost of RATS and the learning curve

The median cost per RATS procedure was £7,167, with theatre costs comprising just over two-thirds of the total cost (NB. an outlier value heavily skewed the postoperative costs; Table 2). The median theatre cost per robotic procedure was £4,606 and the median postoperative cost was £2,035. The two major factors influencing total cost were the OR time and length of hospital stay.

**Table 2:** Costs associated with RATS lung resection.

	ALL RATS (*N* = 365)
Theatre costs	
OR time (in minutes)	2,637 +/− 775 (2540)
Blood transfusion (units of blood)	8 +/− 67 (0)
Staplers	1,264 +/− 0 (1264)
Instrument	467 +/− 0 (467)
Patient drape	10 +/− 0 (10)
Robot drape	250 +/− 0 (250)
Assistant port	48 +/− 0 (48)
Drain	8 +/− 0 (8)
Conversion	97 +/− 455 (0)
*TOTAL Theatre cost*	*4,789 +/*− *1,011 (4606)*
Length of stay	
* General ward*	2,480 +/− 2,684 (1628)
* ICU*	633 +/− 3,415 (0)
Complications	1,606 +/− 2,539 (0)
Re-admission	164 +/− 607 (0)
*TOTAL Postoperative costs*	*4,884 +/*− *7,169 (2035)*
Overall	
TOTAL COST PER PATIENT	9,673 +/− 7,329 (7167)

Mean ± standard deviation (median) per patient. All costs in £GBP. ICU, Intensive care unit; OR, Operating room.

The impact of the learning curve on procedure cost was evaluated. A significantly higher cost during the learning curve could be attributed to theatre time, requirement for blood transfusion and conversion rate (Table 3). The theatre cost per procedure after completion of the learning curve was significantly lower at £4,406 vs. £5,046 pre-learning curve (*p* < 0.001). There was a trend towards increased postoperative costs largely due to significantly increased cost of complications. This could be attributed to a possible selection bias (e.g. increased performance status and cancer stage) secondary to the effect of the COVID-19 pandemic on patient presentation as discussed in more detail below.

**Table 3:** Analysis of cost related to the learning curve. Mean ± standard deviation (median) per patient. All costs in £GBP. OR: Operating room.

	100 EARLIEST RATS (*N* = 101)	100 LATEST RATS (*N* = 101)	*p*-value
Theatre costs			
OR time (in minutes)	3,052 +/− 737 (3000)	2,338 +/− 579 (2360)	<0.001
Blood transfusion (units of blood)	25 +/− 118 (0)	0 +/− 0 (0)	0.024
Staplers	1,264 +/− 0 (1264)	1,264 +/− 0 (1264)	–
Instrument	467 +/− 0 (467)	467 +/− 0 (467)	–
Patient drape	10 +/− 0 (10)	10 +/− 0 (10)	–
Robot drape	250 +/− 0 (250)	250 +/− 0 (250)	–
Assistant port	48 +/− 0 (48)	48 +/− 0 (48)	–
Drain	8 +/− 0 (8)	8 +/− 0 (8)	–
Conversion	200 +/− 639 (0)	0 +/− 0 (0)	0.002
*TOTAL Theatre cost*	*5,323 +/*− *1,113 (5046)*	*4,384 +/*− *579 (4406)*	*<0*.*001*
Length of stay			
*General ward*	2,173 +/− 1,525 (1628)	2,381 +/− 1,679 (2035)	0.350
*ICU*	396 +/− 1,322 (0)	583 +/− 3,618 (0)	0.389
Complications	1,192 +/− 2,185 (0)	2,108 +/− 2,966 (0)	0.012
Re-admission	216 +/− 690 (0)	168 +/− 615 (0)	0.603
*TOTAL Postoperative costs*	*3,978 +/*− *4,288 (2035)*	*5,240 +/*− *6,078 (3621)*	*0*.*053*
Overall			
TOTAL COST PER PATIENT	9,301 +/− 4,688 (7441)	9,624 +/− 6,132 (7481)	0.790

Mean ± standard deviation (median) per patient. All costs in £GBP. ICU, Intensive care unit; OR, Operating room.

### 3.2. Cost of RATS vs. VATS

The cost-effectiveness of RATS and VATS surgery in lung cancer patients was evaluated. Robotic procedures during learning curve were excluded and 101 RATS vs. 101 VATS lung resections were matched and compared. Patient characteristics were similar in both groups (Table 4). Mean operative time was similar in the VATS and RATS groups (115.7 and 115.8 min respectively; *p* = 0.98), as was blood transfusion requirement (0.03 and 0.01 units/case respectively; *p* = 0.52) and number of stapler loads used (8.3 and 8.2 respectively; *p* = 0.88; Table 5). The conversion rate was 4% in the RATS groups and 2% in the VATS group but this was not significantly different. The median length of stay was significantly longer in the RATS group at 5 days vs. 4 days in the VATS group (*p* = 0.025). Complication rate was similar between the two groups (38.6% for RATS and 34.7% for VATS; *p* = 0.433) but the readmission rate was higher in the RATS group (12.9% and 4.0% respectively; *p* = 0.04). All RATS cases for this sub-analysis were performed during the COVID-19 pandemic and 85% of the VATS cases were performed before it.

**Table 4:** Patient characteristics of RATS and VATS lung resections.

	RATS (*N* = 101)	VATS (*N* = 101)	*p*-value
Age*	69.5 +/− 9.1	69.9 +/− 9.0	0.757
Gender (*n*)			
Male	39 (38.6%)	39 (38.6%)	1.000
Female	62 (61.4%)	62 (61.4%)	
Performance Status (ECOG; *n*)			
0	22 (21.8%)	22 (21.8%)	1.000
1	55 (54.5%)	55 (54.5%)	
2	24 (23.8%)	24 (23.8%)	
Smoking status (*n*)			
Non-smoker	20 (19.8%)	13 (12.9%)	0.155
Ex-smoker	59 (58.4%)	72 (71.3%)	
Smoker	22 (21.8%)	16 (15.8%)	
Comorbidity (*n*)			
Pulmonary comorbidity	29 (28.7%)	31 (30.7%)	0.758
Cardiac comorbidity	50 (49.5%)	55 (54.5%)	0.481
Renal comorbidity	6 (5.9%)	6 (5.9%)	1.000
Endocrine comorbidity	18 (17.8%)	17 (16.8%)	0.853
FEV1 (%)*	93.1 +/− 22.2	90.7 +/− 23.6	0.459
TLCO (%)*	75.6 +/− 21.3	74.9 +/− 19.3	0.807
Procedure (*n*)			
Lobectomy	72 (71.3%)	72 (71.3%)	1.000
Bilobectomy	2 (2.0%)	2 (2.0%)	
Segmentectomy	27 (26.7%)	27 (26.7%)	

* Mean ± standard deviation. ECOG: Eastern Cooperative Oncology Group; FEV1: Forced expiratory volume in 1 s; TLCO: Transfer capacity (Lung) for Carbon Monoxide.

**Table 5:** Comparison of theatre and postoperative data from RATS and VATS lung resections.

	RATS (*N* = 101)	VATS (*N* = 101)	*p*-value
Theatre costs			
OR time (minutes)*	115.7 +/− 38.1 (110)	115.8 +/− 25.1 (120)	0.983
Blood transfusion (units per case)*	0.03 +/− 0.3 (0)	0.01 +/− 0.1 (0)	0.528
Staple loads (*n*)*	8.3 +/− 2.4 (8)	8.2 +/− 2.4 (8)	0.883
Conversion rate	4.0%	2.0%	0.683
Other costs			
Length of stay (days)	9.5 +/− 13.9 (5)	6.2 +/− 5.0 (4)	0.025
* Number of days in general ward*	*8.3 +/*− *10.8 (5)*	*5.9 +/*− *4.9 (4)*	*0*.*048*
* Number of days in ICU*	*1.2 +/*− *5.3 (0)*	*0.2 +/*− *0.9 (0)*	*0*.*062*
Complication rate (Clavien-Dindo grade)			
*None*	61.4%	65.3%	0.433
*I*	16.8%	14.9%	
*II*	10.9%	11.9%	
*III*	5.9%	7.9%	
*IV*	2.0%	0%	
*V*	3.0%	0%	
Re-admission rate	12.9%	4.0%	0.040

* Mean ± standard deviation (median) per patient. ICU, Intensive care unit; OR, Operating room.

The overall average cost of a RATS procedure was significantly higher than the cost of a VATS procedure (approximately £1,000/case; *p* = 0.045; Table 6). The relatively high standard deviation associated with postoperative (and hence total) cost in the RATS group, suggests that the average costs are influenced by a few outlier values and so the median values were used for the comparison. Theatre and postoperative costs individually were not significantly different for RATS and VATS lung resections. Of note, stapler cost per case was significantly lower in the RATS group (£1264 vs. £1782; *p* < 0.001).

**Table 6:** Cost comparison of RATS and VATS lung resections.

	RATS (*N* = 101)	VATS (*N* = 101)	*p*-value
Theatre costs			
OR time (in minutes)	2,314 +/− 763 (2200)	2,316 +/− 502 (2400)	0.479
Blood transfusion (units of blood)	5 +/− 49 (50)	2 +/− 17 (0)	0.994
Staplers	1263.6 +/− 0 (1263.6)	1,782 +/− 479 (1738)	<0.001
Instrument	467 +/− 0 (467)	–	–
Patient drape	9.6 +/− 0 (9.6)	9.6 +/− 0 (9.6)	–
Robot drape	250 +/− 0 (250)	–	–
Alexis	–	25 +/− 0 (25)	–
Assistant port	48 +/− 0 (48)	–	–
Port	–	16 +/− 0 (16)	–
Drain	8 +/− 0 (8)	8 +/− 0 (8)	–
Conversion	88 +/− 435	44 +/− 311	0.408
*TOTAL Theatre cost*	*4,456 +/*− *1,075 (4246)*	*4,202 +/*− *885 (4037)*	*0*.*053*
Length of stay (days)			
* General ward*	3,365 +/− 4,377 (2035)	2,410 +/− 2,004 (1628)	0.221
* ICU*	1,361 +/− 5,783 (0)	261 +/− 948	0.974
Complications	1,918 +/− 3,073 (0)	1,358 +/− 1,902 (0)	0.504
Re-admissions	309 +/− 807 (0)	95 +/− 470 (0)	0.023
*TOTAL Postoperative costs*	*6,953 +/*− *11,461 (3256)*	*4,124 +/*− *3,776 (2035)*	*0*.*155*
Overall			
TOTAL COST PER PATIENT	11,406 +/− 11,619 (7467)	8,325 +/−4,153 (6425)	0.045

Mean ± standard deviation (median) per patient. All costs in £GBP. ICU, Intensive care unit; OR, Operating room.

### 3.3. Effect of the COVID-19 pandemic on the cost of RATS

A cost comparison of RATS procedures performed before and during the COVID-19 pandemic was performed on two subgroups of patients (Table 7). The overall cost of procedures performed during the pandemic was similar to those performed before the pandemic (Table 8). Total theatre and postoperative costs demonstrate a paradoxical significant decrease and increase respectively in the during vs. before pandemic groups. Theatre and postoperative costs before the pandemic were £620 more expensive (*p* < 0.001) and £1,221 cheaper (*p* = 0.018) respectively than during the pandemic. A detailed summary of the complications that occurred in the before and during COVID-19 pandemic groups can be found in Supplementary Table S3.

**Table 7:** Demographics of patients undergoing RATS lung resection before and during the COVID-19 pandemic.

	BEFORE COVID (*N* = 171)	DURING COVID (*N* = 194)
Age*	69.7 +/− 10.6 (71)	69.7 +/− 9.1 (71.5)
Gender (*n*)		
Male	59 (34.5%)	73 (37.6%)
Female	112 (65.5%)	121 (62.4%)
Performance Status (ECOG; *n*)		
0	59 (34.5%)	62 (32.0%)
1	80 (46.8%)	96 (49.5%)
2	32 (18.7%)	36 (18.6%)
Procedure (*n*)		
Lobectomy	146 (85.4%)	132 (68.0%)
Bi lobectomy	5 (2.9%)	2 (1.0%)
Segmentectomy	20 (11.7%)	50 (25.8%)
Wedge resection	–	10 (5.2%)
Histology (*n*)		
Adenocarcinoma	110 (64.3%)	116 (59.8%)
Squamous cell carcinoma	26 (15.2%)	23 (11.9%)
Other NSCLC	2 (1.2%)	2 (1.0%)
Metastasis	13 (7.6%)	33 (17.0)
Carcinoid	17 (9.9%)	16 (8.2%)
Other	2 (1.2%)	4 (2.1%)
Adenocarcinoma, SCLC	1 (0.6%)	0 (0%)
Duration of chest drain (days)*	3.6 +/− 5.3 (2)	3.7 +/− 4.7 (2)
Length of stay (days)*	5.6 +/− 4.6 (4)	7.6 +/− 10.6 (5)
Planned ICU admission		
Number of patients	10 (5.8%)	6 (3.1%)
Days in ICU*	1.6 +/− 0.7 (1.5)	2.0 +/− 2.4 (1)
Unplanned ICU admission		
Number of patients	6 (3.5%)	13 (6.7%)
Days in ICU*	8.8 +/− 5.4 (7.5)	9.9 +/− 11.7 (4)
Complications (Clavien-Dindo grade)		
*0*	119 (69.6%)	114 (58.8%)
*I*	18 (10.5%)	37 (19.1%)
*II*	24 (14.0%)	26 (13.4)
*III*	8 (4.7%)	9 (4.6%)
*IV*	2 (1.2%)	4 (2.1%)
*V*	0 (0%)	4 (2.1%)

* mean ± standard deviation (median). ECOG: Eastern Cooperative Oncology Group; ICU: Intensive care unit; NSCLC: Non-small cell lung cancer; SCLC: Small cell lung cancer.

**Table 8:** Effect of the COVID-19 pandemic on cost.

	BEFORE COVID (*N* = 171)	DURING COVID (*N* = 194)	*p*-value
Theatre costs			
OR time (in minutes)	2,932 +/− 730 (2940)	2,377 +/− 720 (2360)	<0.001
Blood transfusion (units of blood)	13 +/− 87 (0)	4 +/− 43 (0)	0.325
Staplers	1,264 +/− 0 (1264)	1,264 +/− 0 (1264)	–
Instrument	467 +/− 0 (467)	467 +/− 0 (467)	–
Patient drape	10 +/− 0 (10)	10 +/− 0 (10)	–
Robot drape	250 +/− 0 (250)	250 +/− 0 (250)	–
Assistant port	48 +/− 0 (48)	48 +/− 0 (48)	–
Drain	8 +/− 0 (8)	8 +/− 0 (8)	–
Conversion	130 +/− 523 (0)	69 +/− 385 (0)	0.196
*TOTAL Theatre cost*	*5,122 +/*− *969 (5026)*	*4,496 +/*− *957 (4406)*	*<0*.*001*
Length of stay			
* General ward*	2,104 +/− 1,509 (1628)	2,811 +/– 3,368 (2035)	0.045
* ICU*	444 +/− 2,092 (0)	799 +/– 4,253 (0)	0.878
Complications	1,259 +/− 2,080 (0)	1,913 +/– 2,855 (0)	0.055
Re-admissions	154 +/− 591 (0)	173 +/– 623 (0)	0.768
*TOTAL Postoperative costs*	*3,961 +/*− *4,529 (1628)*	*5,697 +/*– *8,800 (2849)*	*0*.*018*
Overall			
TOTAL COST PER PATIENT	9,082 +/− 4,784 (7234)	10,193 +/– 8,976 (7099)	0.947

Mean ± standard deviation (median) per patient. All costs in £GBP. ICU, Intensive care unit; OR, Operating room.

## 4. Discussion

In the last two decades the number of lung resections performed through thoracotomy has decreased rapidly (20). Minimally invasive surgery represents the gold standard in the treatment of early stage lung cancer and limited lung metastases. VATS has become the favored approach for anatomical lung resection in respect of better postoperative outcome compared to open surgery with similar long term outcome (2). However, in the last two decades RATS has been introduced with possible advantages including better lymph node dissection, less blood loss, higher complete resection rate, lower conversion rate, less postoperative complications and better quality of life. However, prohibitively high start-up costs and concern regarding ongoing maintenance and disposable costs remain (5).

The median cost of robotic procedures in our institution was £7,167 which is significantly lower than the costs published in a recent systematic review (21, 22). This is likely partly due to our institutions higher case volume (over 700 lung cancer resections per annum) and also the impact of higher costs associated with early robotic experience which these studies describe (12). As with the introduction of most new technologies, during the learning curve the procedure cost is generally higher which is largely attributable to longer operative time. The impact of operative time on the cost of robotic surgery had been documented previously (23). To the best of our knowledge, the present study demonstrates the effect of learning curve on cost of RATS lung resection for the first time. After completing the learning curve, the operative time was shorter and both transfusion requirement and conversion rate were lower with a median saving of £640 per case.

In our series, after completion of the learning curve, VATS and RATS lung resection demonstrated no significant difference in theatre cost as previously reported by Kneuertz et al. (9). Interestingly however, overall cost of RATS vs. VATS was significantly higher largely due to postoperative cost differences. It was necessary to exclude the procedures performed during the learning curve for this analysis, therefore all RATS procedures included in this comparison were performed during the COVID-19 pandemic. However, due to data availability, 85% of the matched VATS procedures took place before the pandemic. Thus, comparisons of the postoperative costs may have been biased by the pandemic which precipitated complex social issues together with reduced support for patients in the community. This in turn increased the length of stay and readmission rate.

The postoperative cost of performing a RATS lung resection was significantly higher (over £1,200/case) during the COVID-19 pandemic vs. before. However, overall costs were comparable due to the significantly reduced theatre costs which occurred in parallel. One might expect the pandemic to increase theatre costs (e.g., with prolonged time needed for aerosol clearance following intubation/extubation). However, the converse was demonstrated in our study. As mentioned previously, the most recent RATS cases were those performed during the COVID-19 pandemic and thus would have been performed after the learning curve had been passed. Therefore, the reduced theatre costs during the pandemic are most likely a reflection of the learning curve process, particularly given the reduction in theatre cost is primarily due to shorter OR time which comes with experience. For this reason, the true extent of the benefit of reduced costs after passing the learning curve in RATS lung resection may be masked in this study. The actual cost savings may be far greater than we report. Furthermore, we demonstrated a significantly reduced stapler cost per case with RATS vs. VATS (approx. £500/case saving). This is most likely due to more frequent fissure dissection with RATS than VATS surgery which saves stapler loads.

The pandemic might be expected to increase postoperative costs and this study confirmed a significant increase of around £1,200/case compared to the pre-pandemic period (*p* = 0.018). This difference is mostly due to a significant increase in the costs associated with length of stay, readmissions and a trend towards an increased cost of complications. In our institution, complications significantly increased in 2020 and 2021 compared to previous years. In-hospital complications were observed in 40.9% of RATS procedures in 2020/2021 compared to the year before (29.7%), a statistically significant increase (*p* = 0.028). Surgical complications increase the total cost of care and this is supported by the findings of this study (24). The reasons for this, highlighted previously include more complex social issues and reduced provision of community support post-discharge.

The strength of this study is that data from a large cohort of patients was prospectively collected and analyzed. The population were homogeneously managed in a single, high volume institution with a detailed and accurate cost analysis. However, the study has several limitations. It is an observational study with a disproportionately high number of robotic procedures performed during the COVID-19 pandemic, which likely introduced bias. Similarly, the cases performed after the learning curve were performed during the pandemic and likely confounded one another. Furthermore, the high capital costs of RATS surgery were not considered. This was largely because the robotic system is also used by other specialties, which makes it challenging to divide these costs accurately between the different specialties.

In conclusion, prior to passing the learning curve, RATS lung resection is associated with increased average procedure cost. When the learning curve is passed, theatre costs become significantly lower and are comparable with VATS. This study likely underestimates the true cost benefit of passing the learning curve due to the effect of the COVID-19 pandemic. The COVID-19 pandemic made RATS lung resection more expensive due to prolonged hospital stay and increased readmission rate. The true postoperative costs of RATS surgery may be similar or even lower than VATS, however further studies are required to elucidate this. The present study offers some evidence that the initial increased costs associated with RATS lung resection may be gradually offset as a program matures.

## Data Availability

The raw data supporting the conclusions of this article will be made available by the authors, without undue reservation.
