# Supplementary material for: A cost analysis of robotic vs. video-assisted thoracic surgery: The impact of the learning curve and the COVID-19 pandemic
Source: Front Surg. 2023 Apr 25;10:1123329. doi: 10.3389/fsurg.2023.1123329 (PMC10167932; doi:10.3389/fsurg.2023.1123329)
Supplement: Supplementary file 1 [file Table1.docx]

Supplementary Material

A cost analysis of robotic vs video-assisted thoracic surgery: the impact of the learning curve and the COVID-19 pandemic

**Oliver J Harrison^*^, Alessandro Maraschi, Tom Routledge, Savvas Lampridis, Corinne LeReun, Andrea Bille**

*** Correspondence:** Oliver J Harrison:ojharrison@doctors.org.uk

# Supplementary Figures and Tables

**Supplementary Table 1 Costing of theatre and postoperative data points.**

| **Item** | **Unit cost** | **Source** |
| --- | --- | --- |
| OR time | £20 per minute | NHS Improvement. Operating Theatres: Opportunities to reduce waiting lists. Understanding theatre productivity metrics. (2019) p7. |
| Blood transfusion | £170 for the first unit  £162 for subsequent ones | NICE Costing statement: Blood transfusion. Implementing the NICE guideline on blood transfusion (NG24). Potential savings, p6,7 (2015) |
| Stapler fires | Covidien stapler for VATS:  £138 for single-use gun  £200 per reload | Guy’s Trust |
|  | Robotic gun  £1263.59 per case | Intuitive Surgical |
| Instrument | £467 per RATS case | Intuitive Surgical (Xi Fen Bipolar £173, Xi Cautery Spatula £173, Xi Cadiere £116) |
| Patient drape | £9.57 per case | Guy’s Trust |
| Robot drape | £250 per RATS case | Guy’s Trust |
| Alexis | £215 per VATS case | Guy’s Trust |
| Assistant port | £48 per RATS case | Guy’s Trust |
| Port | £16 per VATS case | Guy’s Trust |
| Drain | £8 per case | Guy’s Trust |
| Conversion | £2,221 | Rouanet, P., Mermoud, A., Jarlier, M., Bouazza, N., Laine, A. and Mathieu Daudé, H. (2020), Combined robotic approach and enhanced recovery after surgery pathway for optimization of costs in patients undergoing proctectomy. BJS Open, 4: 516-523.  Conversion cost from minimally invasive to open surgery was estimated by multiplying the difference in length of stay between the two surgical approaches (open and minimally invasive) by the cost of stay in the surgical ward and adding to the result the cost of the open surgery materials required in addition to the minimally invasive surgery resources |
| Day in general ward | £407 | NHS National schedule of NHS costs 2018 -2019  Median of national average unit cost across multiple robotic procedure codes calculated by Intuitive |
| Day in ICU | £1,100 | Critical care Tariff  Thoracic surgical adult patients predominate Adult Critical Care, 1 Organ Supported (CCU07-XC06Z) |
| Complications | Grade I: £3,498  Grade II: £3,776  Grade III: £4,916  Grade IV: £11,190  Grade V: £13,611 | Brunelli A et al. The Severity of Complications Is Associated With Postoperative Costs After Lung Resection. Ann Thorac Surg 2017;103:1641–6  Costs were converted into GBP using a conversion rate of 1 GBP = $1.30 |
| Re-admission | £2,400 | NICE National costing statement: Implementing the NICE guideline on Transition between inpatient hospital settings and community or care home settings for adults with social care needs (December 2015)  Average cost per readmission is based on the annual cost to the economy (2.4B) divided by the total number of annual readmissions (1M). Readmissions were defined as all emergency readmissions within 30 days of discharge from an all-cause stay inpatient hospital setting |

Supplementary Table 2 Complications by Clavien-Dindo grade occurring in the RATS lung resection patient cohort.

| **Overview of complications by Clavien-Dindo grade** | **Low-grade (I-II)**  **(N=105)** | **High grade (III-V)**  **(N=27)** |
| --- | --- | --- |
| COVID-19 infection | 0 | 0 |
| Bleeding | 1 | 3 |
| Prolonged air leak (> 7 days) | 25 | 2 |
| Atrial fibrillation | 24 | 5 |
| Atelectasis/sputum plug/bronchoscopy | 12 | 8 |
| Hospital acquired pneumonia | 30 | 11 |
| Pleural effusion/empyema | 3 | 6 |
| Surgical emphysema | 1 | 0 |
| Pneumothorax requiring drain insertion | 3 | 8 |
| Gastrointestinal complication | 16 | 5 |
| Other complication | 14 | 4 |

Supplementary Table 3 Complications by Clavien-Dindo grade that occurred in the RATS lung resection patients before and during the COVID-19 pandemic.

| **Overview of complications by Clavien-Dindo score** | **BEFORE COVID (N=171)** | | **DURING COVID (N=194)** | |
| --- | --- | --- | --- | --- |
|  | **Low-grade (I-II)**  **(N=42)** | **High grade (III-V)**  **(N=10)** | **Low-grade (I-II)**  **(N=63)** | **High grade (III-V)**  **(N=17)** |
| COVID-19 infection | - | - | 0 | 0 |
| Bleeding | 1 | 2 | 0 | 1 |
| Prolonged air leak (> 7 days) | 15 | 1 | 10 | 1 |
| Atrial fibrillation | 8 | 1 | 16 | 4 |
| Atelectasis/sputum plug/bronchoscopy | 4 | 4 | 8 | 4 |
| Hospital acquired pneumonia | 13 | 3 | 17 | 8 |
| Pleural effusion/empyema | 2 | 2 | 1 | 4 |
| Surgical emphysema | 1 | 0 | 0 | - |
| Pneumothorax requiring drain insertion | 0 | 2 | 3 | 6 |
| Gastrointestinal complication | 4 | 1 | 12 | 4 |
| Other complication | 6 | 1 | 8 | 3 |
